# State-dependent capital and income breeding: a novel approach to evaluating individual strategies with stable isotopes

**DOI:** 10.1186/s12983-016-0157-x

**Published:** 2016-06-09

**Authors:** Kim Jaatinen, Markus Öst, Keith A. Hobson

**Affiliations:** Novia University of Applied Sciences, Coastal Zone Research Team, Raseborgsvägen 9, FI-10600 Ekenäs, Finland; Environmental and Marine Biology, Faculty of Science and Engineering, Åbo Akademi University, Artillerigatan 6, FI-20520 Turku, Finland; Environment Canada, 11 Innovation Blvd., Saskatoon, S7N 3H5 Canada; Department of Biology, University of Western Ontario, 1151 Richmond St., London, ON N6A 5B7 Canada

**Keywords:** Resource allocation, Reproductive allocation, Stable isotopes, Capital vs. income, Climate change, *Somateria mollissima*

## Abstract

**Background:**

Species-specific strategies for financing the costs of reproduction are well understood, forming a continuum ranging from high to low reliance on stored nutrients. Animals relying mostly on stored reserves are termed ‘capital breeders’, whereas ‘income breeders’ rely mostly on concurrent intake when financing the costs of reproduction. The role and adaptive value of individual variation in these strategies remain elusive. Life-history theory posits that capital breeding should be favoured when offspring reproductive value peaks, typically occurring early in the season, and that current income should increasingly be used with progressing season. Because resource limitation may hamper flexible resource allocation, a corollary prediction is that only good-condition individuals may show the expected seasonal shift in resource use. To test this prediction, we examined stable isotopes (δ^13^C and δ^15^N) in blood and lipid-free egg yolk of breeding eider females (*Somateria mollissima*) from the Baltic Sea to assess the role of individual variation in the use of proteins from local diet vs. stored reserves.

**Results:**

We show for the first time that individuals from a single population differ in their utilization of stored reserves and concurrent intake to finance the costs of reproduction. Consistent with our prediction, heavy females predominantly used stored reserves for producing egg yolks early in the season, increasingly relying on local feeding with later onset of breeding, whereas light females showed no seasonal change in allocation strategy.

**Conclusions:**

Stable isotope profiling at the individual level is a powerful tool for monitoring relative changes in investment strategies through time, showing promise as an early warning indicator of ecological change in food webs.

## Background

The varying degree to which animals rely on stored reserves and on concurrent intake when financing the costs of reproduction led Drent and Daan [1] to classify bird species as either capital or income breeders based on the cues that determine egg-laying and clutch-size decisions. Later, this dichotomy was extended to a host of animals and plants; reliance on stored resources characterizes capital breeding whereas utilization of concurrent food intake is termed income breeding [2]. The more recent realization that these strategies only represent endpoints of a continuum [3] spurred interest in examining the degree to which species are capital or income breeders and the adaptive value of these strategies [4]. A major discovery in the wake of this research is that capital breeders, particularly migratory ones, often rely more on concurrent intake than previously assumed, presumably because of the costs of carrying excess stores (e.g. [5, 6]). These improvements have moved us beyond the overly simplistic capital-income typology and have increased our understanding of the drivers of this variation in nutrient sources contributing to reproductive investment at the species level. As a result, our knowledge on the relative importance of intrinsic (e.g. individual state) and extrinsic factors in determining the allocation of resources to reproduction is increasing [7]. These studies have considered interspecific comparisons, but we still do not know whether such a capital-income continuum exists among individuals of the same species, nor do we understand the causes of such potential variation.

The optimal allocation between competing life-history functions should depend upon internal state (e.g., condition) and the external environment [8]. Notwithstanding this general understanding, it is striking that our knowledge regarding the sources of energy allocated to reproduction is still in its infancy, despite the isotopic tools being available for decades [9]. To address this knowledge gap, we examined stable isotope values (δ^13^C and δ^15^N) in blood and lipid-free egg yolk of breeding eider females (*Somateria mollissima*) from the Baltic Sea during three years to assess the role of individual variation in the use of proteins from local diet vs. stored reserves.

The eider is especially well suited for the purpose since it was previously deemed an extreme capital breeder [3] which abstains from feeding throughout incubation (e.g. [10]). In the northern Baltic this species is also a short-distance migrant [11], providing ample opportunity to transport stored reserves for breeding from the wintering grounds.

Hobson et al. [5] showed that Baltic eiders produce the yolks of their eggs by allocating approximately half of the proteins from endogenous, stored reserves, and half from local feeding. On the other hand, albumen is produced almost entirely from local resources and only a negligible fraction (0.4-0.7 %) of the proteins stem from endogenous resources. This striking difference in resource allocation between different tissues within an egg is in itself interesting. However, the small fraction of stored reserves used to form albumen allows for very little inter-individual variation in allocation strategies and, as such, albumen tissue is of lesser interest when studying individual variation. Yolks, on the one hand, exhibit more variation in the source of protein from which they are formed due to their relatively greater mass contribution and due to their longer-term development (rapid follicular growth lasts 6-9 days; [12]). In our study population, eiders winter off the Danish coast and arrive on the breeding grounds in Finland with body protein reserves derived from winter diets of Danish mussels. Egg yolks formed from endogenous reserves are isotopically distinct from those that are formed from local diets [5]. However, the degree to which the isotopic signature of the endogenous reserves reflects the diet of the wintering grounds will depend on the time since arrival at the breeding grounds due to natural turnover of body proteins. Elemental turnover rates for carbon in blood are not available for our female eiders (mean mass 2050 g) but a half life of 23 days was estimated for Canvasback (*Aythya valisineria*), another large bodied (~1250 g) diving duck [13]. Applying an allometric correction to the canvasback data derived by Carleton and Martinez del Rio [14] results in a carbon half-life estimate of 27.4 days for eider blood. Thus, the endogenous reserve estimate provided by our model based on eider blood and yolk isotope values will primarily reflect reserves collected on the Danish wintering grounds during the first month following departure from Danish waters. After that the endogenous reserve estimate will progressively represent endogenous reserves formed on the breeding grounds. On the other hand, the contribution of local (exogenous) dietary proteins to egg yolk estimated by our model will not be influenced by time since departure from the wintering grounds. Late breeding females may spend a longer time at the breeding sites, thus filling their reserves with protein from local mussels prior to breeding. Our approach allowed us to disentangle allocation patterns from any potential changes in isotope signature of stored proteins by considering both the timing of breeding and the spatial and isotopic segregation of proteins [15].

In light of the novel evidence suggesting that eiders also utilize local food resources for yolk formation [5], we hypothesized that individuals differ in the degree to which they utilize stored and/or local resources for egg production and energetic needs. We derived specific predictions building upon life-history theory, which holds that the optimal strategy is to first reproduce from both capital and income, and then to rely on current income after storage is used [4]. This is because producing offspring from capital should yield the highest fitness when the reproductive value of offspring peaks, which occurs in the early breeding season in species experiencing a typical seasonal decline in offspring’s reproductive value. The reproductive value of progeny, from the perspective of a parent, can be defined as the likelihood of them surviving to reproductive age and spreading the genes of the parent(s). This value tends to decrease with a progressing season due to increased predation pressure and due to the lesser time available for maturation prior to winter (e.g. [16, 17]). However, we predicted that only females with adequate stored reserves, and hence in good body condition, may be able to flexibly adjust their seasonal resource allocation according to the pattern predicted by theory. In contrast, individuals in poor body condition may depend to a larger extent on locally derived resources (income breeding) throughout the breeding season. To control for variables potentially affecting the energetic demands of breeding, we also related female body size, nest-site cover [18], clutch size and year to the isotopic values of the various tissues examined.

## Results

**Fig. 1:**
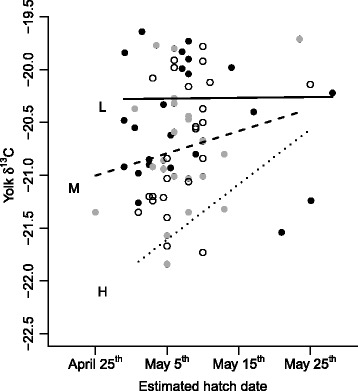
Eider female lipid-free yolk δ^13^C values. Eider female lipid-free yolk δ^13^C values (*N* = 70) are affected by an interaction between timing of breeding and female size corrected weight. Heavy females (75^th^ percentile, dotted line H, open circles) show a transition from utilizing mostly stored reserves (i.e., lower lipid-free yolk δ^13^C values) when breeding early toward an increased use of local feeding (i.e., higher lipid-free yolk δ^13^C values) when breeding later in the season. Light females (25^th^ percentile, solid line L, black dots) do not exhibit an association between timing of breeding and lipid-free yolk δ^13^C values, but mostly utilize proteins gained from local feeding when producing the yolks for their eggs. Females of median weight (dashed line M, grey dots) exhibit an intermediate association between timing of breeding and lipid-free yolk δ^13^C values

**Fig. 2:**
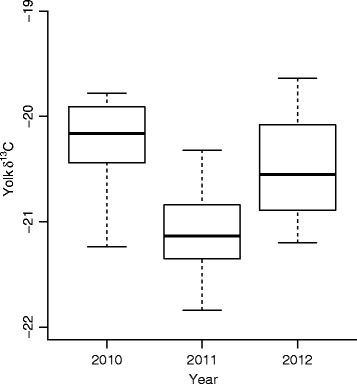
Yearly differences in lipid-free yolk δ^13^C values. Years 2010 (*N* = 21) and 2012 (*N* = 23) differed significantly from 2011 (*N* = 26), but only marginally significantly from one another (whiskers exhibit the range of the data)

The final model explaining lipid-free yolk δ^13^C values was significant and explained 54 % of the variance (*F*_*6 63*_ = 12.57, *p* < 0.0001, *R*^*2*^ = 0.54). This model showed that lipid-free yolk δ^13^C values were explained by an interaction between lay date and size-corrected mass (*b* = 0.029, *F*_*1, 63*_ *=* 11.64, *p* = 0.001; Fig. 1) and differed among years (*F*_*2, 63*_ = 20.57, *p* < 0.0001). The interaction showed that light females relied to a large extent on local protein resources (i.e. they had higher lipid-free yolk δ^13^C), and did not change their allocation patterns as the season progressed. In contrast, heavy females showed a transition from utilizing mostly stored reserves when breeding early toward an increased use of local feeding when breeding later in the season (Fig. 1). Both years 2010 (*z* = -6.25, *p* < 0.0001) and 2012 (*z* = 4.1.9, *p* < 0.0001) differed significantly from 2011, but they differed only marginally significantly from one another (*z* = -2.26, *p* = 0.07) with respect to lipid-free yolk δ^13^C (Fig. 2).

Yolk δ^15^N values were significantly explained only by year, which accounted for 47 % of the variance (*F*_*3, 66*_ = 19.87, *p* < 0.0001, *R*^*2*^ = 0.47). There was, however, a trend-indicating a negative effect of lay date on yolk δ^15^N values (*b* = -0.019, *F*_*1, 66*_ = 3.46, *p* = 0.07). This effect could indicate that late-nesting females utilized more local resources than early nesters (i.e., they had lower yolk δ^15^N values), which is consistent with the positive association between timing of breeding and time spent at the breeding grounds (see below). Years 2010 (*z* = -6.84, *p* < 0.0001) and 2012 (*z* = 5.55, *p* < 0.0001) differed significantly from 2011, but they did not differ from one another (*z* = -1.38, *p* = 0.35) with respect to yolk δ^15^N.

**Fig. 3:**
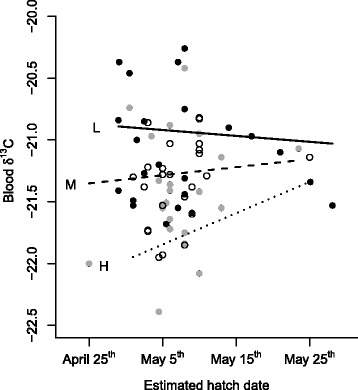
Eider female blood δ^13^C values. Eider female blood δ^13^C values (*N* = 69) are, very similarly to lipid free yolk δ^13^C values, affected by an interaction between timing of breeding and female size corrected weight. Heavy females (75^th^ percentile, dotted line H, open circles) show a clear transition from utilizing more stored reserves (i.e., lower blood δ^13^C values) when breeding early toward an increased use of local feeding (i.e., higher blood δ^13^C values) when breeding later in the season. In contrast, the blood δ^13^C values of light females (25^th^ percentile, solid line L, black dots) are only negligibly affected by the timing of breeding. Females of median weight (dashed line M, grey dots) exhibit an intermediate association between timing of breeding and blood δ^13^C values

The model explaining blood δ^13^C values was significant and explained 24 % of the variance (*F*_*6, 62*_ = 5.77, *p* = 0.007, *R*^*2*^ = 0.24). This model showed that blood δ^13^C values were explained by an interaction between lay date and size-corrected mass (*b* = 0.017, *F*_*1, 62*_ = 4.39, *p* = 0.04; Fig. 3) and differed among years (*F*_*2, 62*_ = 3.58, *p* = 0.03). The interaction is similar to the one observed for lipid-free yolk δ^13^C and showed that the allocation patterns of light females are negligibly affected by the timing of breeding. In contrast, heavy females showed a clear transition from utilizing more stored reserves when breeding early toward an increased use of local feeding when breeding later in the season (Fig. 3). The year 2010 differed from 2011 (*z* = -2.60, *p* = 0.03), but 2012 did not differ from either 2010 (*z* = -0.90, *p* = 0.64) or 2011 (*z* = 1.79, *p* = 0.17).

The final model explaining blood δ^15^N values was significant and explained 28 % of the variance in blood δ^15^N (*F*_*4, 64*_ = 6.29, *p* = 0.0002, *R*^*2*^ = 0.28). This model showed that blood δ^15^N values were significantly explained by year (*F*_*2, 66*_ = 7.78, *p* = 0.0009) and by nest cover (*F*_*2, 64*_ = 3.15, *p* < 0.05). Both years 2010 (*z* = -2.50, *p* = 0.03) and 2012 (*z* = 3.87, *p* = 0.0003) differed significantly from 2011, but 2010 and 2012 did not significantly differ from one another (*z* = 1.28, *p* = 0.41) with respect to blood δ^15^N. The significant effect of nest cover detected by the linear model could not be detected by the post-hoc test, however. This test only detected a trend indicating that females nesting in covered nests utilized more stored protein reserves (i.e., had higher δ^15^N values) than females nesting in open nests (*z* = 2.06, *p* = 0.09). We found no differences between open and semi-covered (*z* = 1.08, *P* = 0.52) or covered and semi-covered nests (*z* = 1.93, *p* = 0.12).

**Fig. 4:**
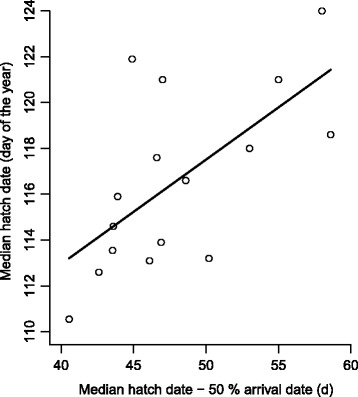
The annual timing of breeding. The annual timing of breeding (median lay date, *N* = 16) of eider females is significantly positively associated with the annual median number of days spent at the breeding grounds prior to egg laying (line represents linear regression: *b* = 0.45, *F*_*1, 14*_ = 10.24, *p* = 0.006, *R* ^*2*^ = 0.42)

The annual median timing of breeding was significantly positively correlated with the annual median number of days spent at the breeding grounds prior to egg laying (*r*_*p*_ = 0.65, *df* = 14, *p* = 0.006; Fig. 4). Interestingly, there was no association between the annual median timing of migration and that of breeding (*r*_*p*_ = 0.06, *df* = 14, *p* = 0.83).

## Discussion

We show for the first time using stable isotope analysis how individuals from a single population differ in the degree to which they utilize stored reserves and concurrent feeding to finance the costs of reproduction. This was made possible by pairing individual physiological and behavioural parameters as well as year effects with individual isotopic measurements of female eiders and their eggs. Our study not only fills in a crucial knowledge gap regarding nutrient allocation strategies, but also shows that unambiguous assignment of species onto the capital-income axis may be difficult. Thus, we were able to show, based primarily on lipid-free yolk and female blood δ^13^C data, that breeding eider females allocate a mixture of local and stored reserves to yolk production. There was also substantial state-dependent individual heterogeneity with respect to the source of nutrients allocated to reproduction (Figs. 1 and 3).

Assuming a seasonal decrease in offspring reproductive value, an assumption that is likely to be met in eiders [19–23], life-history theory posits that capital breeding should boost early reproduction whereas current income should increasingly be used for egg production as the season progresses [4]. We found a weak negative association between lay date and yolk δ^15^N values. The weakness of this association may reflect the context-dependent nature of allocation strategies; consistent with our *a priori* prediction, we found that early breeding heavy females relied almost solely on stored reserves when producing the yolks for their eggs, and to an increasing degree on local feeding as the season progressed (dotted lines in Figs. 1 and 3). Light females, however, relied largely on local feeding when producing yolk and exhibited no change in allocation strategy over the course of the breeding season (solid black line in Figs. 1 and 3), and may actually even slightly increase their reliance on stored reserves over the season (Figs. 1 and 3). This suggests that only females with sufficient reserves at their disposal may be able to pursue a mixed allocation strategy predicted by theory and that females with limited reserves are reliant on a fixed allocation strategy more at the income-breeding end of the capital-income continuum. Furthermore, this finding implies that light and heavy females differ more in their allocation strategies early in the season, but tend to converge in their allocation strategies toward the end of the season (distance between dotted and solid line decreases in Figs. 1 and 3).

The blue mussel is the main prey of eiders across the Baltic Sea (e.g., [24]), but its quality as food, in terms of soft body content, varies considerably over time and space [25, 26], mainly due to climatic fluctuations. During warm winters when seawater temperatures are higher, blue mussels lose mass because of increased metabolism and maintenance costs during times of minimal food intake [26]. Consequently, these mussels are of poor nutritional value for eiders, while cold winters allow mussels to maintain high shell to muscle tissue ratios and so be of high value for feeding eiders [26]. In light of our results, females able to gather large fat stores on the wintering grounds may depend less on the local food source for their own energy budget, whereas females with lesser stores will be more exposed to potential fluctuations in blue mussel stocks at the breeding localities (Figs. 1 and 3). In this respect, it is interesting to note that female body condition is repeatable between years [27] and that females in poor body condition have lower survival prospects [28], for reasons that are unclear but perhaps related to the less flexible breeding resource allocation strategies shown here. The source and amount of proteins and lipids available to eiders and how these macronutrients are allocated between female maintenance through incubation and direct allocations to eggs will reflect, then, the interplay between nutritional conditions and opportunities on both breeding and wintering grounds.

Here, we have considered only protein pathways linking endogenous and exogenous sources to eggs, but δ^13^C analyses are also suited for tracing lipid sources [5]. It is noteworthy that the timing of breeding seems to be more tightly connected to the duration females spend at the breeding grounds prior to initiating breeding (Fig. 4) than to the timing of migration *per se*. This suggests that our finding regarding the seasonal trend in local vs. stored reserves may also have population-level consequences: local food availability and quality may be more important in years when females spend a longer time at the breeding grounds prior to initiating egg laying, whereas in late years that of the wintering grounds may be of more central importance for successful clutch formation and breeding. In this respect, and due to the fact that allocation strategies may differ between different macronutrients [29] future isotopic studies should also investigate the sources of lipids to female energy budgets as well as to eggs.

Climate change is likely to increase the frequency of warm winters that hamper mussel growth and decrease their quality as food for Baltic eiders wintering in the Danish straits [26]. Thereby climate change may lead to greater reliance of eiders on local food resources gathered at the breeding grounds [5], which may in turn result in eiders being forced to breed later (this study; Fig. 4). Delayed breeding may subsequently result in reduced fecundity [19, 22] and fledgling success [21] and thereby reduce the viability of this already endangered population [30]. Thus, stable isotope profiling of breeding animals in general, and eiders in particular, may prove a useful tool for monitoring the impacts of complex, large-scale processes such as climate change on feeding opportunities and breeding propensity, as well as function as an early warning system should long-term changes in resource allocation be detected.

## Conclusions

Our findings show how feeding opportunities on the wintering and breeding grounds are interlinked in female eiders, but also open up the possibility that annual fluctuations in the relative availability of these food sources differentially affect individual fitness depending on state. In light of these novel findings, we urge more research attention to be drawn to individually-based stable isotope studies in order to further our understanding about the causes and consequences of the state-dependent nutrient allocation strategies demonstrated here. Although more research is required to refine our isotopic models, especially those aspects related to isotopic discrimination associated with the mobilization to eggs of endogenous reserves [5], it is clear that we already possess a powerful tool to monitor *relative* changes in female investment strategies through time. Ultimately, consideration of the hitherto unrecognized inter-individual variation in resource allocation patterns may offer a novel approach for elucidating the ramifications of ecological alterations, such as climate change, on food webs.

## Methods

The study was conducted at Tvärminne (59°50’N, 23°15’E), western Gulf of Finland, in 2010–2012. Eiders in this population winter in Danish waters, and their northward migration usually occurs without stopping [11]. Females forage outside the breeding colonies before initiating breeding and during the early stages of laying [31]. Timing of breeding was estimated by measuring the incubation stage using an egg floatation test [18]. Egg sampling was timed to the early phases of incubation. Because laying order may be important in stable isotope studies [32], we collected the darkest egg (visual inspection) – which is typically the first laid egg [33] – from each sampled clutch.

Females were captured during early incubation using hand nets, weighed (to the nearest 5 g), measured for structural size (length of the radius-ulna to the nearest 1 mm) and blood samples were obtained by extracting ~1.5 ml of blood from the ulnar vein. The nest sites of all captured females were classified into one of three classes: open, partly covered or covered [19] and clutch size (prior to egg sampling) recorded. The relative mass estimates were obtained by calculating the standardized residuals from a linear model where log-transformed female mass was explained by log-transformed size and by the log-transformed number of days since the start of incubation. A relative mass estimate was used to allow comparison of females of all sizes. One egg was collected for stable isotope analysis from each nest upon capture of the female. Egg sampling and female handling procedures, including ringing, were approved by the Animal Experiment Board/State Provincial Office of Southern Finland, number ESLH-2009-02969/Ym-23, and Tvärminne Zoological Station.

When eiders arrive in spring to their breeding grounds they may need to wait for the remaining snow and ice to melt on and around the breeding islands as well as for the ground to dry sufficiently as not to jeopardize egg viability. Variation in the length of this waiting period at the breeding grounds may affect the amount of stored reserves left for egg formation. To estimate the period spent at the breeding grounds prior to initiating breeding, we utilized daily migration counts of migrating eiders collected in 1997-2012 at the Hanko Bird Observatory (HALIAS) situated 20 km west of Tvärminne. The time spent at the breeding grounds prior to breeding was given by the number of days between the arrival of the 50^th^ percentile of the population at HALIAS [34] and the median annual estimated lay date at Tvärminne from the corresponding time period (1997-2012).

The isotope analysis protocol, mixing model details and the endogenous and exogenous endpoint calculations used have been recently described in detail by Hobson et al. [5]. All stable isotope values are reported in delta (δ) notation as the parts per thousand deviation in ratios of heavy to light isotope abundance in samples vs. those of international standards (Vienna PeeDee Belemnite for δ^13^C and AIR for δ^15^N). Briefly, we considered protein source contributions from endogenous (modelled on female blood isotope values) and exogenous (local blue mussel, *Mytilus edulis*) endpoints and applied isotopic discrimination factors linking lipid-free egg yolk isotope values with these two sources. These discrimination factors were based on a captive eider study [35] for exogenous (i.e. local dietary routing) pathways and the carnivore model of Hobson [36] for the endogenous (i.e. mobilization from stored proteins) pathway [37]. Mixing models to estimate relative contributions from the two sources were run using MixSIR (v. 1.0, [38]) for each individual. Our previous study showed that eggs formed from endogenous protein reserves had higher δ^15^N and lower δ^13^C values than those formed from exogenous sources [5]. We could thus interpret individual strategies of female nutrient investment into eggs based on relative egg δ^15^N and δ^13^C values and how these changed through time.

Values of δ^13^C and δ^15^N for female blood and lipid-free egg yolk were explained by year, clutch size, lay date, female size-corrected mass, female size and nest cover as well as all their two-way interactions (except those with year) in separate generalized linear models (GLMs). Only three of the 76 females sampled over the three-year study period were sampled in two different years, thus precluding the necessity of including female identity as a random effect in the models. Due to the few years of data, year was treated as a categorical variable in the analysis. Because of this, each individual year will require one degree of freedom, which is why interactions between year and other covariates were omitted, to ensure an acceptable ratio of sample size to covariates in model selection. Female mass declines with advancing incubation and this decline may mask any potential effect of size-corrected mass on isotope values in egg components. Incubation stage was hence included in all models in which size-corrected mass was present. The presence of multicollinearity between explanatory variables was assessed by calculating the variance inflation factors (VIFs). No multicollinearity was detected and all VIFs were below 1.80. All non-significant variables were dropped from the models in a step-wise manner based on their F-value calculated from Type II sums of squares, which are unaffected by the order of covariates. The final model only contained significant variables. Significant interactions were graphically illustrated using simple slope analysis [39], by estimating the slope of the independent variable on the dependent variable at high (75th percentile), median, and low (25th percentile) levels of the moderator. If significant year effects were detected, the years were compared with one another using multiple comparisons of means based on Tukey’s contrasts.

Pearson correlation coefficients were calculated to examine associations between the timing of breeding and both the period spent at the breeding grounds prior to egg laying and the timing of migration.

The residuals of all models adhered to the assumption of independence and normality. All analyses were performed using the R 3.1.2 software [40].

## Abbreviations

HALIAS, Hanko bird observatory; VIF, variance inflation factor
